# Integrating single-cell transcriptomics and machine learning to reveal prognostic and immune significance of lactylation in breast cancer

**DOI:** 10.1016/j.gendis.2026.102099

**Published:** 2026-02-16

**Authors:** Da Qian, Haotian Liu, Jianchen Mou, Zhuotao Yang, Liquan Zhu, Chaoqi He, Bo Wang, Xuli Meng

**Affiliations:** a Central Laboratory, Changshu Hospital Affiliated to Soochow University, Changshu No. 1 People's Hospital, Changshu, Jiangsu 215500, China; b Cancer Center, Department of Breast Surgery, Zhejiang Provincial People's Hospital (Affiliated People's Hospital), Hangzhou Medical College, Hangzhou, Zhejiang 310014, China; c China Key Laboratory for Diagnosis and Treatment of Upper Limb Edema and Stasis of Breast Cancer, Hangzhou, Zhejiang 310014, China; d Department of Thyroid and Breast Surgery, Changshu Hospital Affiliated to Soochow University, Changshu No. 1 People's Hospital, Changshu, Jiangsu 215500, China

Breast cancer (BC), the second leading cause of female death worldwide, keeps rising in incidence and remains difficult to cure.^1^ Despite advances in screening and therapy, many patients still suffer a poor prognosis; novel prognostic biomarkers and predictive models are urgently needed to guide individualized treatment. Discovered in 2019, lactylation attaches lactate to lysine via lactoyltransferases/delactylases, modulating proteins in hypoxia, immunity, and tumorigenesis.^2^ Linked to poor prognosis in gastric cancer, liver cancer, and other cancers, its role in BC biology, microenvironment, and therapy remains poorly understood, urging integrative models for diagnosis and treatment. In this pioneering study, we revealed, for the first time at the single-cell level, the complex connection between elevated lactylation levels and BC development and progression. We employed a combination of multiple machine learning models to construct a robust lactylation-related gene (LRG) risk scoring model at the single-cell level, establishing it as an independent prognostic factor. This lactylation risk score, derived through machine learning, accurately reflects the lactylation levels at the single-cell transcriptome level. This risk score has been validated with external datasets. Our findings reveal its role in predicting prognosis, providing mechanistic insights, and identifying potential therapeutic pathways for BC. The flowchart is shown in Figure 1.

**Figure 1:**
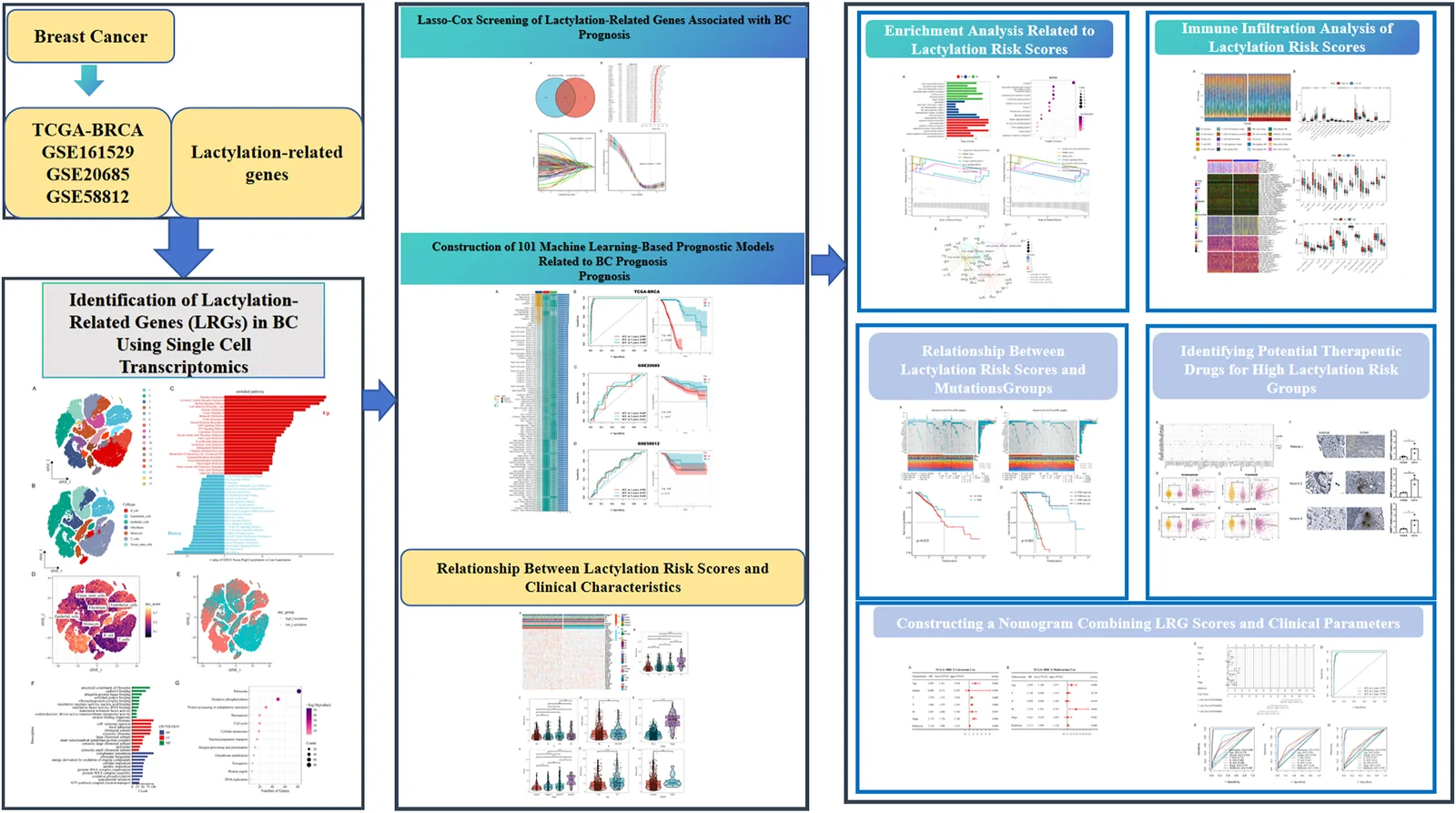
The flowchart.

First, to identify lactylation-associated genes, we analyzed single-cell RNA-sequencing data from 48,961 QC-filtered cells: t-SNE resolved 18 clusters (Fig. S1A) and t-SNE mapped six major subpopulations (Fig. S1B). The preprocessing of single-cell RNA-sequencing data in our study was performed using the Seurat R package, following its standard workflow. Gene Set Variation Analysis (GSVA) revealed pathways enriched in high-versus low-lactylation groups (Fig. S1C). “AUCell” package in R, a widely used and well-established tool for evaluating gene set activity at the single-cell level, was employed to calculate lactylation activity scores. The gene set representing lactylation was curated based on known lactylation regulators and substrates reported in the literature. Area under curve (AUC) scores for lactylation signatures peaked in immune cells, notably B cells (Fig. S1D). Cells were dichotomized by median AUC into high/low-lactylation groups (Fig. S1E). Differential analysis yielded 1437 differential expression genes; intersection with AUC-correlated genes expanded the list to 4214 lactylation-associated genes. Gene Ontology/Kyoto Encyclopedia of Genes and Genomes (GO/KEGG) enrichment highlighted oxidative phosphorylation, estrogen receptor (ER) protein processing, and cell cycle (Fig. S1F and S1G). In summary, we catalogued 1437 LRGs in BC.

Intersecting 1437 differential expression genes with 4214 AUC-linked genes yielded 861 high-confidence lactylation genes (Fig. S2A). Univariate Cox analysis of TCGA-BC identified 138 prognostic candidates (*P* < 0.05) (Fig. S2B). We also adopted LASSO-Cox regularization, where the default settings in the glmnet R package were used. 10-fold cross-validation were applied, and the dataset was randomly split into training and test sets, typically in a 7:3 ratio, which retained 37 optimal genes: AIMP1, ANAPC11, C8orf33, CBR1, CD24, CEBPD, CHORDC1, CLDN7, CLTA, COX7B, EIF3H, EIF4G1, EMP1, GLUL, GRPEL1, KDELR2, KLRB1, MAL2, MAT2A, MMP7, MRPL18, NONO, PIGR, PNN, PPIB, PSMB1, RPA3, RPL14, RPL19, RPL38, RPS24, RTN4, SFPQ, SH3BP5, SNX9, TANK, and VDAC1 (Fig. S2C and S2D).

As shown in Figure S3A, a total of 101 prognostic models were obtained. StepCox[forward]+RSF achieved the highest mean C-index (0.736) across TCGA-BRCA, GSE20685, and GSE58812, so its 37-gene signature was selected. RiskScore = Σ(coefᵢ × exprᵢ). Patients were stratified by the optimal cut-off; high-risk patients showed worse overall survival in all cohorts: TCGA 1/3/5-y AUC = 0.983/0.989/0.989; GSE20685 = 0.649/0.699/0.661; GSE58812 = 0.582/0.631/0.646 (Fig. S3B–S3D).

Heatmap revealed LRGs up-regulated in high-risk BC, correlating with worse clinicopathology (Fig. S4A). Risk scores differed significantly across survival status, age (> 65 *vs*. ≤ 65), T, N, and Stage (*P* < 0.05; Fig. S4B–S4H), indicating escalating scores parallel deteriorating clinical features.

Differential analysis between risk groups identified 169 genes. GO showed chemokine binding, cytokine activity, immunoglobulin complex, and humoral immunity (Fig. S5A). KEGG showed cytokine/chemokine and T-cell signaling (Fig. S5B). Gene Set Enrichment Analysis (GSEA) showed FoxO, cancer, antigen presentation, estrogen, and PPAR pathways (Fig. S5C and S5D). GSVA showed E2F targets, G2M checkpoint, and mTORC1 (Fig. S5E). These immune-centric changes link risk stratification to T-cell biology in BC.

The CIBERSORT (Fig. S6A) showed higher infiltration of macrophages in the high-LRG score group, while B cells, T cells, and natural killer (NK) cells showed lower infiltration (Fig. S6B). High-risk tumors exhibited elevated checkpoint, cytolytic, and type-II-IFN responses (Fig. S6E). Six algorithms confirmed robust correlations between LRG risk scores and immune infiltration (Fig. S6C), underscoring lactylation-driven tumor microenvironment remodeling as a potential immunotherapy target in BC.

Mutational landscapes (Fig. S7A and S7B) and survival curves showed that high tumor mutational burden portends worse overall survival (Fig. S7C). Patients with high lactylation risk and high tumor mutational burden exhibited the poorest survival, whereas low lactylation risk and low tumor mutational burden had the best (Fig. S7D). Thus, lactylation risk scores correlate with tumor mutational burden and may predict mutation load and immune responsiveness in BC.

Drug-sensitivity profiling showed that MMP7 and KLRB1 negatively correlated with IC50 (Fig. S8A). Low-risk tumors were more sensitive to doramapimod, trametinib, and oxaliplatin, whereas high-risk tumors responded better to lapatinib (Fig. S8B–S8E). Immunohistochemistry assays confirmed MMP7 overexpression in BC (Fig. S8F). Thus, MMP7/KLRB1 are candidate immunotherapy targets, and lapatinib is a rational choice for high-lactylation-risk BC. Matrix metalloproteinases (MMPs) are important enzymes that play a role in the invasion and metastasis of tumors by acting on the extracellular matrix. MMP7 plays a key role in tumor invasion and metastasis and has been explored as a therapeutic target in several cancers. In the meantime, current findings suggest that MMP7 can increase the incidence of BC in smokers and alcohol users.^3^ KLRB1 encodes CD161, which is a C lectin-like receptor, primarily expressed on NK cells and T cells. A very recent and important study has shown that it can regulate immune cell activity, and its high expression is generally associated with more active anti-tumor immunity and better prognosis.^4^ In a lactylation-reprogrammed tumor microenvironment, expression levels of MMP7 and KLRB1 and their function may be influenced by metabolic and immune context, suggesting value as an immune-related drug response marker.

Multivariate analysis confirmed LRGR as an independent prognostic factor (Fig. S9A and S9B). A nomogram integrating LRGR with key clinicopathologic variables accurately predicted 1-, 3-, and 5-year overall survival in TCGA-BRCA (Fig. S9C). The areas under the receiver operating characteristic (ROC) curves (AUCs) reached 0.991, 0.991, and 0.990, respectively (Fig. S9D), outperforming any single clinical feature (Fig. S9E–S9G), providing a robust tool for personalized BC management.

BC is the second most common female malignancy, yet its heterogeneity and complex tumor microenvironment limit traditional therapies.^5^ Once viewed as waste, lactate now drives immune evasion and resistance via lactylation. Although lactylation signatures have been reported in lung, liver, and gastric cancers, a BC-specific profile is lacking.

Here, we integrated single-cell data to select 861 lactylation regulators and bulk RNA sequencing from 1049 BC patients to retain 138 prognostic genes. LASSO-Cox distilled 37 key LRGs, with StepCox+RSF yielding the top-performing model (mean C-index: 0.736). A 37-gene risk score stratified 434 external patients (derived from TCGA datasets GSE20685 and GSE58812) into high- and low-risk groups with robust 1-, 3-, and 5-year AUCs (0.98–0.99). High scores correlated with advanced age, T/N/Stage, and poor overall survival. Functional enrichment highlighted cytokine-chemokine, PPAR, and FOXO pathways; most LRGs were FOXO-down-regulated, suggesting PI3K/AKT/ERK-mediated control. Immune profiling revealed high-risk tumors enriched in macrophages but depleted in B, T, and NK cells. Drug-sensitivity screens linked MMP7 and KLRB1 to lapatinib benefit in high-risk cases and to doramapimod, trametinib, and oxaliplatin in low-risk cases. A nomogram combining the score with clinical variables outperformed standard predictors (AUC ≈ 0.99).

Limitations include scarce subtype analyses, absence of functional assays, and limited lactylation antibodies. Future proteomic and mechanistic studies are warranted to validate these 37 LRGs as therapeutic and immunotherapeutic targets in BC.

## CRediT authorship contribution statement

**Da Qian:** Writing – review & editing, Writing – original draft, Visualization, Formal analysis, Data curation, Conceptualization. **Haotian Liu:** Data curation. **Jianchen Mou:** Writing – original draft, Methodology, Investigation, Formal analysis, Data curation. **Zhuotao Yang:** Visualization, Validation, Investigation. **Liquan Zhu:** Visualization. **Chaoqi He:** Visualization, Supervision. **Bo Wang:** Writing – review & editing, Supervision, Project administration, Funding acquisition. **Xuli Meng:** Writing – review & editing, Supervision, Project administration, Funding acquisition, Conceptualization.

## Ethics declaration

This study was approved in writing by the Ethics Committee of Zhejiang Provincial People's Hospital (No. 20241011725429), and the patient's tumor tissue was obtained after an informed consent form was provided by the patient. All procedures were conducted in accordance with the ethical standards of the responsible committee on human experimentation and with the Helsinki Declaration of 1975, as revised in 2000.

## Data availability

The data are available by emailing the corresponding author.

## Funding

This research was supported by the 10.13039/501100001809National Natural Science Foundation of China (No. 82404685), the “Pioneer” and “Leading Goose” R&D Programs from 10.13039/501100017599Zhejiang Science and Technology Department (China) (No. 2023C03044), and the 10.13039/501100018556Suzhou Science and Technology Development Program (China) (No. SKYD2023087).

## Conflict of interests

The authors declared no conflict of interests.
