# Supplementary material for: Integrating single-cell transcriptomics and machine learning to reveal prognostic and immune significance of lactylation in breast cancer
Source: Genes Dis. 2026 Feb 16;14(3):102099. doi: 10.1016/j.gendis.2026.102099 (PMC13718854; doi:10.1016/j.gendis.2026.102099)
Supplement: Supplementary file 1 [file mmc1.docx]

**Materials and methods**

1. **Data Sources**

The single-cell RNA sequencing (scRNA-seq) data for breast cancer used in this study was obtained from the GSE161529 database (https://www.ncbi.nlm.nih.gov/geo/), which includes eight BC samples. The bulk RNA-seq data of the training set BC was sourced from The Cancer Genome Atlas (TCGA) BRCA cohort (https://portal.gdc.cancer.gov/repository). After excluding cases with missing survival time and incomplete clinical information, a total of 1049 BC samples were included. The validation sets were obtained from the Gene Expression Omnibus (GEO) database of the National Center for Biotechnology Information (NCBI) (https://www.ncbi.nlm.nih.gov/geo/), with GSE20685 comprising 327 patients and GSE58812 comprising 107 patients. Based on previous literature reports, 332 lactylation-related genes were selected. Supplementary Table 1.

2. **scRNA-seq Data Processing and Identification of Lactylation-Related Genes (LRGs)**

The "Seurat" package in R was used to analyze the scRNA-seq data. During the quality control stage, cells expressing 200-7000 genes in at least three cells, with mitochondrial genes not exceeding 10%, were selected to ensure high-quality single-cell data. After normalization using the "ScaleData" function, 48961 cells were obtained. Data integration was performed using the "FindClusters" and "FindNeighbors" functions to determine cell clusters, and cell annotation was based on marker genes (Supplementary Table 2, 3, 4). Cellular heterogeneity was assessed, and the "AUCell" function in R was used to calculate lactylation activity scores, which were then assigned to individual cells. Subsequently, cells were divided into high-lactylation and low-lactylation AUC groups based on the median AUC score, and visualized using the "ggplot2" package. Differential analysis was then conducted to identify differentially expressed genes (DEGs) between the high-lactylation and low-lactylation groups, leading to further exploration of 1437 DEGs (Supplementary Table 5). Additionally, correlation analysis was performed to identify genes strongly associated with lactylation activity. The intersection of correlated genes and DEGs was used as target genes (Supplementary Table 6) for future studies. DEGs and CORGs represent genes that significantly influence lactylation activity (resulting in 861 genes after intersection) (Supplementary Table 7). To explore the biological functions of differential genes between high and low lactylation groups, we used the "clusterProfiler" package in R to perform Gene Ontology (GO) and Kyoto Encyclopedia of Genes and Genomes (KEGG) analyses, identifying the biological pathways involving candidate genes (Supplementary Table 8, 9). Additionally, we used GSVA to analyze the biological processes involving candidate genes in the Hallmark pathways.

**3. Lasso-Cox Related Gene Screening**

The 861 genes screened from scRNA-seq were subjected to univariate Cox analysis to identify genes related to patient survival. To avoid overfitting, Lasso (least absolute shrinkage and selection operator) regression was used. We used the default settings in the glmnet R package for LASSO Cox regression, with 10-fold cross-validation to determine the optimal regularization parameter (lambda), the dataset was randomly split into training and test sets, typically in a 7:3 ratio, and genes with non-zero coefficients were considered potential prognostic genes.

**4. Machine Learning Risk Scoring Construction**

To construct a stable lactylation-related gene prognostic model, the prognostic genes identified by Lasso were incorporated into a comprehensive machine learning model, including Random Survival Forest (RSF), Elastic Net (Enet), Lasso, Ridge, StepCox, CoxBoost, Cox partial least squares regression (plsRcox), Supervised Principal Component (SuperPC), Generalized Boosted Regression Model (GBM), and survival Support Vector Machine (survival-SVM). On the TCGA-BRCA dataset, a leave-one-out cross-validation (LOOCV) framework was used to fit predictive models through 101 algorithm combinations for prognostic markers and validated on the GSE20685 and GSE58812 datasets. For each model, the Harrell's concordance index (C-index) was calculated for the training set TCGA-BRCA and validation sets GSE20685 and GSE58812, and the model with the highest average C-index was considered optimal. Similar machine learning algorithms have been reported in previous studies. Based on the median risk score of TCGA-BRCA patients, the TCGA-BRCA, GSE20685, and GSE58812 datasets were divided into high-risk and low-risk groups, and survival curves for the high- and low-risk groups were plotted using the "survminer" package in R.

5. **Relationship Between Lactylation Risk Scores and Clinical Characteristics**

To explore the relationship between lactylation risk scores and clinical characteristics of breast cancer, we used the Wilcoxon test to compare the differences in risk scores across different clinical characteristics and visualized the results using box plots with "ggplot". A heatmap was also generated to display the distribution of clinical characteristics and risk genes.

6. **Enrichment Analysis of Differential Genes Between High and Low-Risk Groups**

To explore the molecular significance of transcriptomic and genetic differences between high- and low-risk groups and to gain insight into the biological processes associated with poor survival in high-risk groups, differential analysis was conducted, identifying 169 genes with differential expression between the high- and low-risk groups. GO analysis revealed that these genes were mainly involved in immune-related biological processes such as chemokine receptor binding, cytokine activity, immunoglobulin complex, and humoral immune response. KEGG analysis showed that these genes were primarily active in cytokine and chemokine signaling pathways, Human T cell-related immune pathways. Further GSEA analysis revealed that these genes were significantly enriched in pathways such as FoxO signaling pathway, Pathways in cancer, Antigen processing and presentation, Estrogen signaling pathway, and PPAR signaling pathway. Additionally, GSVA analysis of candidate genes in Hallmark pathways identified a significant enrichment of genes in lactylation-related pathways such as HALLMARK_E2F_TARGETS, HALLMARK_G2M_CHECKPOINT, and HALLMARK_MTORC1_SIGNALING. Overall, these genes are involved in the immune biology of BC patients, suggesting a strong correlation between high and low-risk groups and patient immunity, particularly in T cells.

**7. Immune Cell Infiltration Analysis**

In this study, we estimated the level of immune cell infiltration in the tumor microenvironment using seven algorithms: "CIBERSORT," "MCP counter," "EPIC," "ESTIMATE," "TIMER," "quanTiseq," and "IPS." Visualization was performed using the "ComplexHeatmap" package. Specifically, we used the R package CIBERSORT and single-sample Gene Set Enrichment Analysis (ssGSEA) to determine the distribution of immune cell types and the enrichment scores of each immune cell between the high-risk and low-risk groups.

8. **Predicting Response to Immunotherapy**

To evaluate the potential differences in treatment response between the high-risk and low-risk groups, we also collected immunotherapy data from the IMvigor210 dataset (urothelial carcinoma, UC). In this dataset, we calculated the IRG risk score to predict the response to immunotherapy.

**9. Mutation Landscape Analysis of High and Low-Risk Groups**

To compare genetic mutations between high and low-risk groups and explore the impact of different mutation burdens on BC patient survival, the gene mutation profile of BC patients was downloaded from the TCGA database. The "maftools" package was used to generate the gene mutation profile of BC patients, and the risk score files were combined to plot waterfall charts and survival curves.

**10. Drug Sensitivity Analysis**

Using the "oncPredict" package in R^,^ the half-maximal inhibitory concentration (IC50) values of drugs for each BC patient were predicted. The differences in IC50 values between high-risk and low-risk groups were tested using the Wilcoxon test, and scatter plots were generated using "ggplot2". Additionally, Spearman's correlation was calculated between gene expression levels in the model and drug IC50 values.

**11. Immunohistochemistry**

Human BC tissues and normal tissues were obtained from Zhejiang Provincial People's Hospital (Affiliated People’s Hospital). The immunohistochemical analysis was processed according to the instructions of the IHC Kit (ImmunoWay, USA, RS0064) to detect MMP7 (Santa Cruz Biotechnology, USA, sc-80205) expression.

**12. Nomogram Model Construction**

For better clinical application, we conducted univariate and multivariate Cox regression analyses to evaluate the independent prognostic value of lactylation gene risk scores, determining whether lactylation gene risk scores are independent prognostic factors for predicting BC patient survival. To further improve the prognostic accuracy and predictive capability of our model, we created a nomogram combining risk scores, age, T stage, N stage, M stage, and Stage as independent prognostic criteria to calculate the probability of 1-year, 3-year, and 5-year OS. The predictive discrimination and accuracy of the nomogram were assessed using ROC curves.

**13. Statistical Analysis**

For normally distributed variables in two comparison groups, we applied an unpaired Student's t-test to determine statistical significance. If the variables followed a non-normal distribution, the Mann-Whitney U test was used to assess statistical significance between the two groups. For comparisons of more than two continuous variables, we used parametric methods such as ANOVA and non-parametric methods like the Kruskal-Wallis test. To explore linear relationships, Pearson or Spearman correlation analyses were conducted between variable pairs. Kaplan-Meier survival analysis was used to compare overall survival rates between high-risk and low-risk groups, and the log-rank test was used to evaluate differences in survival curves between the two groups. Additionally, ROC curves were generated, and AUC values were calculated to evaluate the predictive performance of the risk scores. All statistical analyses were performed using R software (version 4.3.1). Data are expressed as means ± SD, with statistical significance set at P < 0.05.
