# Supplementary material for: Integrating single-cell transcriptomics and machine learning to reveal prognostic and immune significance of lactylation in breast cancer
Source: Genes Dis. 2026 Feb 16;14(3):102099. doi: 10.1016/j.gendis.2026.102099 (PMC13718854; doi:10.1016/j.gendis.2026.102099)
Supplement: Supplementary file 2 [file mmc2.docx]

**Figure S1: Identification of Lactylation-Related Genes in BC Patients Using Single Cell Transcriptomics. A, B:** t-SNE plots showing the annotation of seven different cell types within the TME of BC samples. As shown, each color on the plot corresponds to a specific cell type. **C:** GSVA bar chart sorted by t-value shows pathways related to the high-lactylation group and low-lactylation group. **D:** AUC scores represent lactylation abundance in individual cells, with brighter colors indicating higher scores. **E:** Lactylation activity of each cell, with orange representing the high-lactylation group and blue representing the low-lactylation group. **F:** Visualization bar chart for Gene Ontology (GO). **G:** Visualization dot plot for Kyoto Encyclopedia of Genes and Genomes (KEGG).

**Figure S2: Screening of Lactylation-Related Genes Associated with BC Prognosis. A:** Venn diagram of differentially expressed genes and highly lactylation-related genes between high and low-lactylation groups. **B:** Forest plot showing the top 30 BC patient prognostic genes selected by univariate Cox analysis among the 138 genes. **C, D:** Lasso-Cox algorithm screened 36 potential BC prognostic genes.

**Figure S3: Construction of Multiple Machine Learning Risk Scoring Models. A:** Machine learning C-index heatmap of training set TCGA-BRCA and test sets GSE20685 and GSE58812. **B:** ROC curve and K-M survival curve of the TCGA-BRCA cohort. **C:** ROC curve and K-M survival curve of the GSE20685 cohort. **D:** ROC curve and K-M survival curve of the GSE58812 cohort.

**Figure S4: Relationship Between Lactylation-Related Genes and Risk Scores. A:** Heatmap of model gene expression levels in different clinical characteristics. **B-H:** Box violin plots showing differences in lactylation risk scores across T stage, N stage, M stage, survival status, Stage stage, age, and sex.

**Figure S5: Enrichment Analysis of Differential Genes Between High and Low-Risk Groups**. **A:** Visualization bar chart for GO enrichment analysis. **B:** Visualization dot plot for KEGG enrichment analysis. **C, D:** GSEA enrichment analysis of upregulated and downregulated pathways. **E:** Related chart for GSVA enrichment in Hallmark pathways.

**Figure S6: Immune Infiltration Analysis of Lactylation Risk Scores. A:** Landscape of immune cell infiltration across different patients calculated using the CIBERSORT algorithm. **B:** Significant differences in immune cell infiltration between high and low-risk groups calculated using the CIBERSORT algorithm. **C:** Differences in immune infiltration status between different risk groups assessed by five algorithms. **D, E:** Significant differences in immune cell infiltration and immune function between high and low-risk groups calculated using the ssGSEA algorithm.

**Figure S7: Relationship Between Lactylation Risk Features and Mutations. A:** Waterfall chart of the top 20 mutated genes in high-risk BC patients. **B:** Waterfall chart of the top 20 mutated genes in low-risk BC patients. **C:** OS survival curve comparing high and low mutation groups. **D:** Kaplan-Meier curve combining risk scores and TMB in BC patients.

**Figure S8: Relationship Between Lactylation Scores and Drug Sensitivity.** A: Scatter plot showing the correlation between lactylation risk gene expression levels and drug IC50 values. **B-E:** Box plot and correlation plot showing differences in IC50 values of Doramapimod, Trametinib, Oxaliplatin, and Lapatinib between high and low lactylation risk groups. **F：**Three patients' images of MMP7 immunohistochemistry (50 µm) and relative expression between normal and tumor. Data are presented as mean ± SD. * p < 0.05, ** p < 0.01.

**Figure S9: Constructing a Nomogram Combining Lactylation Risk Scores and Clinical Characteristics. A, B:** Univariate and multivariate Cox regression analysis shows that lactylation risk scores are independent prognostic factors. **C:** Nomogram combining risk scores and clinical characteristics. **D:** ROC curve for predicting 1, 3, and 5-year outcomes using the nomogramodel. **E-G:** ROC curves comparing the predictive performance of the nomogram with other clinical features.
